# The Effects of Iron Oxide Nanoparticles Administration on Depression Symptoms Induced by LPS in Male Wistar Rats

**DOI:** 10.29252/NIRP.BCN.9.3.209

**Published:** 2018

**Authors:** Fatemeh Saeidienik, Mohammad Reza Shahraki, Hamed Fanaei, Fereshteh Badini

**Affiliations:** 1. Department of Physiology, School of Medicine, Zahedan University of Medical Sciences, Zahedan, Iran.; 2. Pregnancy Health Research Center, Zahedan University of Medical Sciences, Zahedan, Iran.; 3. Department of Biology, Faculty of Sciences, Payame Noor University, Tehran, Iran.

**Keywords:** Depression, Iron nanoparticle, Antidepressive effect, Lipopolysaccharide

## Abstract

**Introduction:**

Depression is a mood disorder in which feelings of sadness, loss, anger, or frustration interfere with everyday life for one to several weeks. Several studies have shown that iron nanoparticles have neuroprotective and anti-inflammatory effects. This study aimed to evaluate anti-depressive effect of iron nanoparticles in male rats.

**Methods:**

Depression was induced by Lipopolysaccharide (LPS) adminstration. Rats were randomly assigned into six groups (10 in each group): 1) control (sterile saline solution; 200 μL, IP); 2) LPS (LPS;100 μg/kg, IP); 3) Low dose Iron Nanoparticle (LINP) (1 mg/kg, IP); 4) High dose Iron Nanoparticle (HINP), 5 mg/kg IP); 5) LPS/LINP (LPS; 100μg/kg IP+INP 1 mg/kg IP); and 6) LPS/HINP (LPS; 100 μg/kg IP+INP 5 mg/kg IP). All injections were performed every other day. To assess the effect of iron nanoparticles on depression symptoms, rats were subjected to two behavioral tests: Forced Swim Test (FST) and Open Field Test (OFT).

**Results:**

Iron nanoparticles treatment in 1 mg/kg and 5 mg/kg doses groups significantly improved depression symptoms when assessed by OFT and FST. In OFT, the number of line crossings, entrance to central square, rearing and duration of attending in central square increased after iron nanoparticles adminstration in depressed rats. Iron nanoparticles adminstration reduced immobility time confirmed by FST and OFT. Also, iron nanoparticles adminstration significantly increased duration of swimming in FST depressed rats.

**Conclusion:**

Our results for the first time showed potential advantageous effect of iron nanoparticles administration in attenuating depression symptoms, which was possibly mediated by modulation of neurotransmitters and anti-inflammatory effects of iron nanoparticles.

## Highlights

- Depression is a mood disorder in which feelings of sadness, anger, or frustration interfere with everyday life for one to several weeks.
- Several studies have shown that iron nanoparticles have neuroprotective and anti-inflammatory effects.
- This study aimed to evaluate anti-depressive effect of iron nanoparticles in male rats.
- Results of the present study showed beneficial effects of iron nanoparticles administration in attenuating depression symptoms.
- These effects may be mediated by modulation of neurotransmitters and anti-inflammatory effects of iron nanoparticles.

## Plain Language Summary

- Depression is a common diseases associated with sadness, anger, or frustration. It affects people’s lives and family relationships, as well as their employment. Iron is one of the essential elements for carrying out activities of human cells and its deficiency can cause disorders. Evidence has shown that iron has protective effects on the nervous system. The use of nanotechnology has created a new hope for the treatment of diseases. Thanks to nanotechnology, medications can reach the target tissue faster and easier. Regarding the beneficial effects of iron for the nervous system, iron nanoparticles were used to treat depression in rats. In this study, the effects of iron nanoparticles on the behavioral patterns such as motor activity and frustration were investigated using laboratory tools. Iron nanoparticles increased motor activity and reduced the sense of frustration in animals. It is believed that the disruption of the neurotransmitter system of the brain or increased inflammation is involved in the development of depressive illness. Thus, iron nanoparticles may have beneficial effects on depressive symptoms by modifying the neurotransmitter system and reducing inflammation. In conclusion, iron nanoparticles can open a new way to treat depression.

## 1. Introduction

Nanoparticles has brought promising hope for the treatment of various diseases (Archakov, 2010). The unique properties of nanoparticles include their greater solubility in lipids, passing more easily through the blood-brain barrier, and greater reactivity (Archakov, 2010). These features are suitable for their use in many medical and biological fields (Berry & Curtis, 2003). Iron is an essential element for living organisms and plays an important role in biological functions (Beard et al., 2005). Iron has various roles in nerve cells, which can be applied to the oxide reductase enzymes, absorption and transmission of neurotransmitters, the brain’s myelin formation and metabolism (Beard, Erikson, & Jones, 2002; Delima et al., 2005; Beard, 2001). Also iron is required for the synthesis of brain neurotransmitters such as serotonin, dopamine, norepinephrine, and gamma-aminobutyric acid (Elseweidy &Abd El-Baky, 2008). Evidence has shown that 25% iron supplementation improves stress and depression in mothers with iron deficiency (Beard et al., 2005). Today depression is a serious disorder in the world, so that WHO predicts that it will be the second most common cause of disability by 2020 (Cryan & Mombereau, 2004). Depression is a mood disorder in which feelings of sadness, loss, anger, or frustration interfere with everyday life for one to several weeks (Kuo, Tran, Shah, & Matorin, 2015; American Psychiatric Association, 2013).

New advances in the theory of major depression have shed lights over the roles of inflammatory processes and immune responses in the pathogenesis of depression (Raison, Capuron, & Miller, 2006; Mössner et al., 2007; Schiepers, Wichers, & Maes, 2005). Researchers believe that chronic inflammation can contribute to the development of depression (Celano & Huffman, 2011). Several studies have shown that iron nanoparticles have neuroprotective and anti-inflammatory effects (Lee, Shin, Lee, & An, 2014). Thus, in this study we assessed anti-depressive effect of iron nanoparticles in male rats.

## 2. Methods

### 2.1. Animals and treatments

This study was approved by the Institutional Animal Research Ethics Committee of Zahedan University of Medical Sciences, Zahedan, Iran. All chemicals were obtained from Sigma-Aldrich (America), unless otherwise stated. A total of 60 male Wistar rats (weighing 200–250 g) were used in the present study. Animals were maintained under 12 hour lighting cycle at room temperature and humidity controlled vivarium. Animals permitted ad libitum access to water and standard lab chow.

Rats were randomly assigned into one of six groups (10 in each group): 1. control (sterile saline solution, 200 μL IP); 2. LPS (LPS; 100 μg/kg IP); 3. Low dose Iron Nanoparticle (LINP) (1 mg/kg IP); 4. High dose Iron Nanoparticle (HINP); 5. mg/kg IP), LPS/LINP (LPS; 100μg/kg IP+INP 1 mg/kg IP); 6. LPS/HINP (LPS; 100μg/kg IP+INP 5 mg/kg IP). All injections were performed every other day.

Escherichia coli Lipopolysaccharide (LPS), and iron nanoparticle dissolved in sterile saline (0.9% NaCl) were injected Intraperitoneally (IP). Control animals were injected with saline. Details of assessment and characteristics of iron oxide nanoparticle are: Iron Oxide Nanopowder (gamma- Fe _2_ O _3 _), Purity: >99.5%, Color: red brown, APS: 20 nm, SSA: 40–80 m ^2^ /g, Morphology: spherical.

### 2.2. Behavioral tests

Rats were subjected to two behavioral tests: Forced Swim Test (FST) and Open Field Test (OFT).

### 2.3. Open field test

OFT was used to assess spontaneous motor activity (Belviranlı & Okudan, 2015). OFT setting consisted of a apparatus with black square box (80 cm × 80 cm) with 40 cm high walls. Animals were placed in the box and their movements were assessed over a 5-min period. Following parameters were recorded: number of squares crossed, entrance to center square, urination, defecation, rearing, stretching, and time of immobility.

### 2.4. Forced Swim Test (FST)

FST was performed according to the method of Martin Seligman. A vertical glass cylinder (50 cm high, 40 cm in diameter) was filled with 23°C water to a depth of 35 cm. For testing, each rat was placed in the cylinder for 5 min, and duration of immobility, swimming, and climbing were scored. Water in the cylinder was changed for each rat. Immobility was recorded whenever animals stopped swimming and remained floating in the water, with their heads above the surface.

### 2.5. Statistics analysis

All data were presented as mean±SEM and analyzed using SPSS version 24. Comparisons were evaluated by 1-way analysis of variance (ANOVA) test followed by Tukey’s multiple comparison tests. P<0.05 was considered statistically significant.

## 3. Results

### 3.1. Effects of iron oxide nanoparticles on open field test results

**Figure 1:**
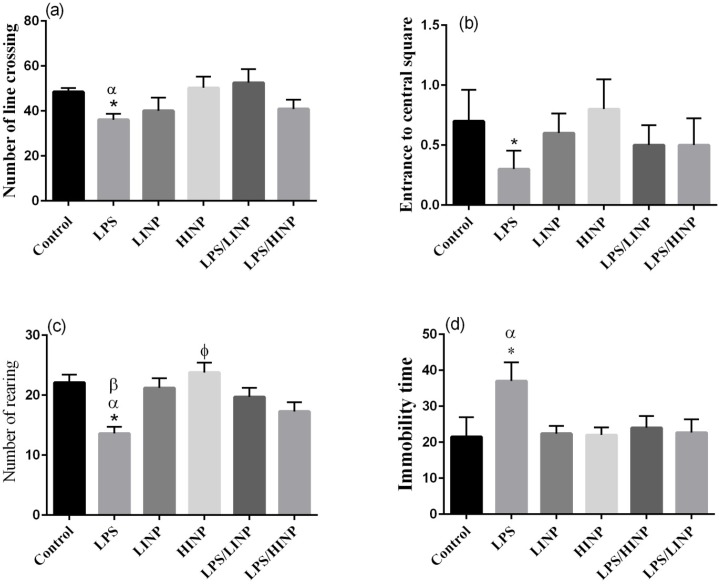
Effect of 1 mg/kg and 5 mg/kg iron nanoparticles administration on (a) number of line crossing, (b) entrance to central square, (c) rearing and (d) immobility time after LPS administration in OFT Values are presented as mean ± SEM (n= 10 per group). *P<0.05 LPS vs. LPS/LINP and HINP, α P<0.01 LPS vs. Control; *P<0.05 LPS vs. Control, LPS/LINP, LPS/HINP, HINP and LINP; *P<0.01 LPS vs. Control and LINP, α P<0.05 LPS vs. LPS/LINP, βP<0.001 LPS vs. LPS/HINP and HINP, Φ P<0.05 HINP vs. LPS/HINP; *P<0.05 LPS vs. Control and HINP, α P<0.01 LPS vs. LINP and LPS/HINP.

As shown in Figure 1a, number of line crossing in LPS group was significantly lower than that in the control, LPS/LINP and HINP group (P<0.01, P<0.05, P<0.05) (F _5,54_ =2.76). The number of entrance to the central square in the control, LPS/LINP, LPS/HINP, HINP and LINP group was significantly higher than that in LPS group (P<0.05) (F _5,54_ =2.43) (Figure 1b).

Number of rearing behavior (Figure 1c) in LPS group was significantly lower than that in the control, LINP, LPS/LINP, LPS/HINP and HINP group (P<0.01, P<0.01, P<0.05, P<0.001, P<0.001) (F _5,54_ =6.18). Number of rearing behavior in HINP group was significantly lower than that in LPS/HINP group (P<0.05) (F _5,54_ =6.18). Duration of immobility in LPS group was significantly longer than that in the control, HINP, LINP and LPS/HINP group (P<0.05, P<0.05, P<0.01, P<0.01, respectively) (F _5,54_ =2.49) (Figure 1d).

**Figure 2:**
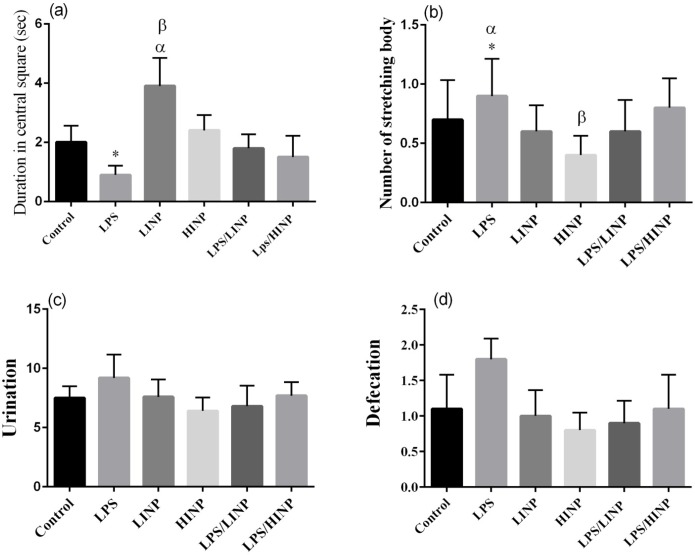
Effect of 1 mg/kg and 5 mg/kg iron nanoparticles administration on (a) duration of staying in central square, (b) number of stretching body, (c) urination and (d) defecation after LPS administration in OFT Values are presented as Mean±SEM. (n=10 per group). *P<0.01 LPS vs. LPS/LINP, α P<0.05 LINP vs. Control, β P<0.05 LINP vs. LPS/LINP; *P<0.05 LPS vs. control and LINP, α P<0.05 LPS vs. LPS/LINP, βP<0.05 HINP vs. LPS/HINP.

As shown in Figure 2, duration of attending in central square of LPS group was significantly shorter than that in the control group (P<0.01) (F _5,54_ =2.71). Duration of attending in central square of LINP group was significantly longer than that in the control and LPS/LINP group (P<0.05) (F _5,54_ =2.71). Number of stretching body in LPS group was significantly higher than those in LINP, LPS/LINP group (P<0.05) (F _5,54_ =2.58). Number of stretching body in LPS/HINP group was significantly higher than that in the HINP group (P<0.05) (F _5,54_ =2.58).

### 3.2. Effect of iron oxide nanoparticles on forced swim test results

**Figure 3:**
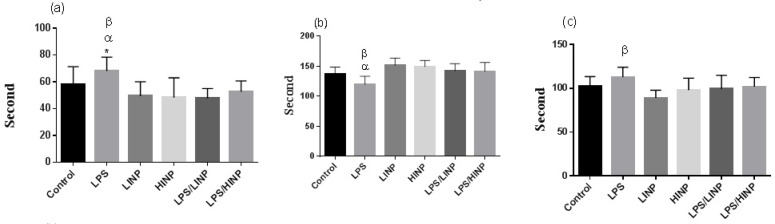
Effect of 1 mg/kg and 5 mg/kg iron nanoparticles administration on duration of (a) immobility, (b) swimming and (c) climbing in FST Values are presented as Mean±SEM (n=10 per group). (a) *P<0.05 LPS vs. LPS/HINP, α P<0.001 LPS vs. LPS/LINP, LPS/HINP and LINP; (b) α P<0.001 LPS vs. LPS/LINP and LPS/HINP, , βP<0.0001 LPS vs. HINP and LINP; (c) β P<0.0001 LPS vs. LINP.

Result of FST has shown that the duration of immobility in this test was significantly higher in LPS group than control, LPS/HINP, LPS/LINP, LINP and HINP group (P<0.05, P<0.05, P<0.01, P<0.01, P<0.01, respectively) (F _5,54_ =5.11). Duration of immobility in control group was significantly higher than that in the HINP group (P<0.05) (F _5,54_ =5.11) (Figure 3).

Duration of swimming in LPS/LINP and LPS/HINP groups were significantly longer than that in LPS group (both P<0.001) (F _5,54_ =8.46). Also, duration of swimming in LPS group was significantly shorter than that in HINP and LINP groups (both P<0.0001) (F _5,54_ =8.46). Duration of climbing in LPS group was significantly longer than LINP group (P<0.05) (F _5,54_ =4.07).

## 4. Discussion

Results of the present study showed that iron nanoparticles have beneficial effects in reducing depression symptoms. In the present study, we induced depression by LPS administration in rats and assessed their symptoms by open field and forced swim tests. LPS administration had detrimental effect on behavioral test results. Simultaneously, iron nanoparticles injections in both 1 mg/kg and 5 mg/kg improved values in open field and forced swim tests.

Iron element has important role in oxygenation of brain parenchyma and thus allows normal function of the brain (Młyniec, Davies, de Agüero Sánchez, Pytka, Budziszewska, & Nowak, 2014). In addition, iron is the element required for energy production, normal neuronal cell function, neurotransmitter synthesis, and myelination (Dusek, Jankovic, & Le, 2012). Thus, brain iron levels have strong effects on behaviors (Kim & Wessling-Resnick, 2014). Several mechanisms are suggested for the role of iron in behavior regulation (Kim & Wessling-Resnick, 2014). Iron deficiency results in attenuation of myelination and monoamine metabolism (Kim & Wessling-Resnick, 2014). Glutamate and GABA metabolism is altered by fluctuations in brain iron status. Such changes lead to emotional and psychological problems. Iron acts as a cofactor for tyrosine hydroxylase and tryptophan hydroxylase enzymes and in this way participates in dopamine and serotonin synthesis (Kim & Wessling-Resnick, 2014). These neurotransmitters are involved in the regulation of mood, neuronal activity, and anxiety (Kim & Wessling-Resnick, 2014). Also, human studies showed iron supplementation in patients with depression has beneficial effects and improves their symptoms (Młyniec et al., 2014; Kim & Wessling-Resnick, 2014).

Depression is a heterogeneous mental disorder with symptoms such as hopelessness, anxiety, irritability, lack of motivation, and feelings of guilt (Murray & Lopez; 1997). WHO predicts that depression will reach the second most prevalent disease by 2020 (Murray & Lopez; 1997). Various nano-sized particles have been applied as nano-enabled drug-delivery systems (Xie, Wang, Zhang, Ren, & Yang; 2012). By crossing the blood brain barrier, they provide novel approaches to treat brain diseases. Formulation of drugs in nano-size enhances the efficiency of drug administration to brain targets, such as release of neurotransmitters, delaying progression and or treating neurodegenerative diseases (Xie et al., 2012) .

Iron element is involved in many physiological functions of the body. This element spreads throughout the body into different shapes. Human studies showed that about 50% of the women all over the world are iron deficient (Beard et al., 2005). The daily iron requirement of a woman (aged between 19 and 50 years) is 18 mg/d (Beard et al., 2005). Iron deficiency in the body lead to anemia. Research indicates a significant relationship between anemia and depression. In other words, a negative correlation between hemoglobin concentration and depression are shown (Beard et al., 2005). Also, iron deficiency in women increases the risk of postpartum depression (Beard et al., 2005), i.e. reduction in blood serum ferritin levels would exacerbate the condition. Iron is also associated with some brain functions (Etebary, Nikseresht, Sadeghipour, & Zarrindast, 2010). Iron is an essential cofactor in the production of most neurotransmitters as well as myelination in CNS. Inactivity increases iron loss, which induces feeling of confusion and lethargy (Etebary et al., 2010).

In the present study, the effect of iron oxide nanoparticles on behaviors like depression induced by lipopolysaccharide was studied. The results of this study suggested that iron oxide nanoparticles improve the condition of depressed patients. OFT and FST are usually adapted for diagnosis and evaluation of depressive-like behaviors (O’Connor et al., 2008; Bouwknecht, Spiga, Staub, R., Hale, Shekhar, & Lowry, 2007). Results obtained in this study showed that rats of LPS group have longer durations of immobility in FST and OFT and lower numbers of crossings and rearing in OFT. Our finding are similar to the results reported by other studies. Abilities were severely impaired in LPS group. Such behaviors as exploring the surrounding environment and emotions that could emulate and assent with clinical symptoms of depression (Xie et al., 2012). Iron nanoparticles administration alleviate symptoms in rats treated with LPS.

Similarly, Youngling et al. reported that rats treated with LPS had lower number of crossings and rearing behavior in OFT and also longer duration of immobility in TST and FST (Xie et al., 2012). Our study shows that iron oxide nanoparticles have antidepressant effects. The above results agree with other studies results conducted on the usual mix of iron or studies on depression and anxiety as a result of iron deficiency. For example, iron deficiency alters the activity and physical reactions such as increased depression-like behaviors (Beard et al., 2005). Also, it has been shown that iron supplementation improves stress and depression in patients with 25% iron deficiency (Beard et al., 2005).

Results of our study support benefits of using iron oxide nanoparticles for treating depression symptoms. Based on the results of our study and other similar studies, iron oxide nanoparticles can be used as an alternative for anti-depressant drugs in treating depressive symptoms. Finally, we suggest doing more studies to evaluate the effects of iron oxide nanoparticles on neurotransmitters and inflammatory factors involved in depression.

## Ethical Considerations

### Compliance with ethical guideline

This study was approved by the Institutional Animal Research Ethics Committee of Zahedan University of Medical Sciences, Zahedan, Iran.
